# Plasma MMP-9 and TIMP-1 levels on ICU admission are associated with 30-day survival

**DOI:** 10.1007/s00508-019-01592-x

**Published:** 2020-01-13

**Authors:** Galateja Jordakieva, Roswitha M. Budge-Wolfram, Alexandra C. Budinsky, Mariam Nikfardjam, Georg Delle-Karth, Angelika Girard, Jasminka Godnic-Cvar, Richard Crevenna, Gottfried Heinz

**Affiliations:** 1 Department of Physical Medicine, Rehabilitation and Occupational Medicine, Medical University of Vienna, Währinger Gürtel 18–20, 1090 Vienna, Austria; 2 Division of Angiology; Department of Internal Medicine II, Medical University of Vienna, Währinger Gürtel 18–20, 1090 Vienna, Austria; 3 International Hospital Development & Hospital Management, Abu Dhabi, United Arab Emirates; 4 Department of Laboratory Medicine, Medical University of Vienna, Währinger Gürtel 18–20, 1090 Vienna, Austria; 5 Department of Cardiology and Intensive Care, Wilhelminen Hospital Vienna, Vienna, Austria; 6 Department of Cardiology, Vienna North Hospital, Vienna, Austria; 7 Division of Cardiology/Intensive Care Unit 13H3; Department of Internal Medicine II Medical, Medical University of Vienna, Währinger Gürtel 18–20, 1090 Vienna, Austria

**Keywords:** Matrix metalloproteinases, Tissue inhibitors of matrix metalloproteinases, SAPS II, Critically ill patients, Survival

## Abstract

**Background:**

Matrix metalloproteinases (MMPs) are involved in systemic inflammatory responses and organ failure. The aim of this study was to evaluate early circulating plasma levels of MMP‑2, MMP‑9 and their inhibitors TIMP‑1 and TIMP‑2 and their prognostic significance in critically ill patients on admission to the intensive care unit (ICU).

**Methods:**

In a single center prospective study 120 consecutive patients (72.5% male, mean age 66.8 ± 13.3 years, mean simplified acute physiology score [SAPS II] score 52.9 ± 21.9) were enrolled on transfer to the ICU of a cardiology department. The most common underlying conditions were cardiac diseases (*n* = 42.5%), respiratory failure (*n* = 10.8%) and sepsis (*n* = 6.7%). Blood samples were taken within 12 h of ICU admission. The MMP‑2, MMP‑9, TIMP‑1 and TIMP‑2 levels in plasma were evaluated in terms of 30-day survival, underlying condition and clinical score.

**Results:**

On ICU admission 30-day survivors had significantly lower plasma MMP‑9 (odds ratio, OR 1.67 per 1 SD; 95% confidence interval, CI 1.10−2.53; *p* = 0.016) and TIMP‑1 (OR 2.15 per 1 SD; 95% CI 1.27−3.64; *p* = 0.004) levels than non-survivors; furthermore, MMP‑9 and TIMP‑1 correlated well with SAPS II (both *p* < 0.01). In patients with underlying cardiac diseases, MMP‑9 (*p* = 0.002) and TIMP‑1 (*p* = 0.01) were independent predictors of survival (Cox regression). No significant correlation was found between MMP‑2 and TIMP‑2 levels, MMP/TIMP ratios and 30-day mortality.

**Conclusion:**

The MMP‑9 and TIMP‑1 levels are significantly elevated in acute critical care settings with increased short-term mortality risk, especially in patients with underlying heart disease. These findings support the value of MMPs and TIMPs as prognostic markers and potential therapeutic targets in conditions leading to systemic inflammation and acute organ failure.

## Introduction

Matrix metalloproteinases (MMPs) are a group of zinc-dependent endopeptidases, which have the ability to disintegrate proteins of the extracellular matrix (ECM) [1]. They support migration of immune cells to infection sites and are further involved in a variety of endogenous proinflammatory and vasoactive cytokine responses, as well as in coagulation and fibrinolysis cascades [2, 3]. The activity of these enzymes is essential in several physiological processes, such as growth and wound healing but also in inflammatory and vascular pathophysiology (e.g. tissue remodeling, arteriosclerosis) [2]. Their activity is regulated in vivo by the specific endogenous tissue inhibitors of metalloproteinases (TIMP) [4–6]. The balance of MMPs and TIMPs is important in order to maintain the integrity of the extracellular matrix [5].

### MMPs and TIMPs in critical illness and cardiovascular disease

The inflammatory response following severe injury or infection is known to be regulated by a complex network of cytokines, initiated by tumor necrosis factor alpha (TNF-alpha). In most underlying conditions that lead to a fatal outcome critically ill patients seem prone to a systemic inflammatory cytokine storm [7]. Among the enzymes secreted by leukocytes, MMPs and their inhibitors are induced and involved in this systemic inflammatory cascade [8–10]; particularly MMP‑9 has been shown to cleave inactive TNF‑alpha into its active form [11]. The MMPs and their inhibitors have been associated with mortality in organ injury [12–14] and sepsis [15], where serum levels of TIMP‑1 and TIMP-1/MMP‑9 ratios have been associated with disease severity and mortality [16–18].

The gelatinases MMP‑2 and MMP‑9 have a central role in cardiovascular disease [19, 20]; they are reportedly associated with heart tissue remodeling and were shown to be increased in the failing myocardium [5, 21–23]. As an enzyme responsible for myocardial fibrosis [22], MMP‑2 may even be more sensitive than established markers, such as the brain natriuretic peptide (BNP) in heart failure patients with preserved ejection fraction [24]. Furthermore, MMP‑2 was shown to play a role in the oxidative stress response after ischemia/reperfusion injury [25] and is also proposed as a biomarker of cardiovascular remodeling in hypertension and left ventricular (LV) dysfunction [20]. In the early phase of myocardial infarction, MMP‑9 release is stimulated, as it cleaves mediators derived from neutrophils and dying cardiomyocytes [21]. The MMP‑9 plasma levels are elevated in coronary heart disease [26, 27] and associated with atherosclerotic plaque destabilization [28, 29], acute coronary events [30, 31] and mortality [32] in these patients. Accordingly, MMP‑9 was shown to be up-regulated during heart failure [33]. The inhibitors of MMP‑2 and MMP‑9, TIMP‑1 and TIMP‑2 are elevated in tissue and plasma during heart failure and myocardial infarction [5, 20, 22, 34, 35]. Higher circulating MMP‑9 and TIMP‑1 levels and MMP-9/TIMP‑1 ratios were reported in ischemic cardiomyopathy [36] and are associated with disease severity in heart failure [37] and tissue remodeling in myocardial hypertrophy [38]. The TIMP‑1 was further shown to correlate with infarct size in ST-elevation myocardial infarction [39]. Both MMP‑2 and MMP‑9 are involved in several cardiovascular and inflammatory diseases; they have been previously associated with critical disease progression and organ injury; however, their predictive value in a heterogeneous group of critically ill patients has not yet been evaluated. Thus, the rationale behind this study was to 1) determine the levels of MMP‑2, MMP‑9 and their inhibitors TIMP‑1 and TIMP‑2 in critically ill patients on admission to ICU, 2) evaluate the potential prognostic significance of MMPs and TIMPs on mortality and further 3) correlate MMP and TIMP levels with other laboratory parameters and an approved clinical survival score (simplified acute physiology score, SAPS II) in these patients.

## Patients, material and methods

### Patient population, study protocol and follow-up

The study was performed in accordance with the Declaration of Helsinki (1964), including current revisions, the Austrian Drug Law (*Arzneimittelgesetz* 1996) and the good clinical practice (GCP) guidelines of the European Commission. Approval from the ethics committee of the Medical University of Vienna (*Ethik Kommission der Medizinischen Universität Wien*) was obtained before initiating the study (Trial registration number: 5982006). The design was a prospective, single center observational study conducted in 120 consecutive patients admitted to the ICU of an internal medicine department, which is mainly dedicated to treat cardiology patients but also admits patients after open heart and thoracic surgery and comprises the entire spectrum of medical patients with critical illnesses.

### End point

End point was death at 30 days. Death was defined as all causes of mortality.

### Blood sampling

In all patients blood samples for determination of MMP‑2, MMP‑9, and their specific inhibitors (TIMP‑1 and TIMP-2) were taken within 12 h after admittance to the ICU. All samples were separated immediately and samples were frozen at −80 °C and stored for not longer than 6 months until analysis.

### Laboratory tests

Stored plasma was analyzed for MMP‑2, MMP‑9, and their specific inhibitors TIMP‑1 and TIMP‑2 with commercially available ELISA (enzyme-linked immunosorbent assay) according to the manufacturer guidelines (Human, Biotrak ELISA System, Amersham, Biosciences, Freiburg, Germany), and TNF-alpha (Human TNF-alpha/TNFSF1A Quantikine, R&D System Inc., Abingdon, UK). The following routine laboratory tests were performed: renal and liver profile, complete blood count, coagulation and infection parameters.

### Primary analysis

Evaluation of prognostic significance of MMP‑2, MMP‑9, and their specific inhibitors (TIMP‑1 and TIMP-2) on ICU admission in an unselected population and evaluation of plasma levels of MMP‑2, MMP‑9, and their specific inhibitors (TIMP‑1 and TIMP-2) on discharge from ICU or when primary endpoint was met (mortality).

### Secondary analyses

Evaluation of prognostic significance of MMP‑2, MMP‑9, and their specific inhibitors (TIMP‑1 and TIMP-2) in relation to SAPS II scores and cofactors, markers of inflammation, TNF-alpha levels, infection (y/n), shock, surgery (y/n), semiquantitative and quantitative notation especially on the basis of left ventricular function, ICU survival, in-hospital survival, presence of coronary heart disease, acute myocardial infarction (y/n) as basic diagnosis, and diagnosis at time of admission.

### Statistical analysis

Analysis was performed with SPSS 20.0 (SPSS, Chicago, IL, USA). A probability value <0.05 was considered as statistically significant and MMPs were examined as continuous variables. Continuous variables were expressed as mean ± standard deviation or median and range if the assumption of a normal distribution was violated. Categorical variables were expressed as counts and percentages. Groups were then compared by Student’s t‑test or Mann-Whitney U‑test, as appropriate. Receiver operating characteristic (ROC) curves were generated using 30-day survival as a classification variable and MMP, TIMP and SAPS II as prognostic variables. The SAPS II, MMP‑9 and TIMP‑1 were evaluated for their independent association with hospital survival by logistic regression. Cox proportional hazard analysis was used to determine an association between 30-day mortality and MMP levels. Univariate logistic regression analysis was used to estimate odds ratios associated with an increase per standard deviation (OR per 1‑SD) of measurements. Multivariate analysis (logistic regression) was performed to take into account the effect of possible confounders. All variables that showed baseline imbalances (*p* < 0.1) between 30-day mortality and MMP levels or according to MMP levels were included in the multivariate model. The 30-day mortality according to the MMP‑2, MMP‑9 and TIMP‑1, TIMP‑2 levels was evaluated by calculating Kaplan Meier estimates.

## Results

### Patients

Altogether, 120 patients (87 male, 33 female, age 66.8 ± 13.3 years) were consecutively enrolled in this prospective single center observational study. The mean SAPS II was 52.9 ± 21.9. Patients’ primary diagnoses on admission and clinical characteristics are described in Tables 1 and 2.

**Table 1:** Primary diagnoses at ICU admission

	All patients (*n* = 120)	Alive (*n* = 96)	Deceased (*n* = 24)	*p*-value^a^
Cardiac disease, *n* (%)	51 (42.5)	37 (72.5)	14 (27.5)	0.168
Cardiac surgery, *n* (%)	26 (21.7)	25 (96.2)	1 (3.8)	
Cardiogenic shock, *n* (%)	5 (4.2)	4 (80.0)	1 (20.0)	
Sepsis, *n* (%)	8 (6.7)	6 (75.0)	2 (25.0)	
Respiratory failure, *n* (%)	13 (10.8)	9 (69.2)	4 (30.8)	
Other diseases, *n* (%)	17 (14.2)	15 (88.2)	2 (11.8)	

*P*-value refers to significance level of differences in 30-days survival (alive/deceased) between subgroups of primary diagnoses (Mann Whitney U test).

*p* ≤ 0.05 was considered statistically significant

^a^*p*-value alive vs. deceased

**Table 2:** Patient characteristics

	All (*n* = 120)	Alive (*n* = 96)	Deceased (*n* = 24)	*p*-value^C^
Male *n*, (%)	87/120 (72.5%)	71/96 (74.0%)	16/24 (66.7%)	0.474
Age, years	66.8 ± 13.3^b^	66.0 ± 13.7^*b*^	70.3 ± 11.0^b^	0.151
SAPS II	*52.9* *±* *21.9*^*b*^	*47.6* *±* *18.3*^*b*^	*74.2* *±* *22.4*^*b*^	*<0.001*
MMP‑2, ng/ml	1223.2 (1107.0)^a^	1186.8 (839.6)^a^	1926.1 (1318.6)^a^	0.126
MMP‑9, ng/ml	*183.7 (259.1)*^a^	*182.9 (235.3)*^a^	*250.8 (344.1)*^a^	*0.009*
TIMP‑1, ng/ml	*310.6 (347.0)*^a^	*302.3 (269.0)*^a^	*575.7 (611.2)*^a^	*<0.001*
TIMP‑2, ng/ml	44.9 (19.9)^a^	44.8 (19.5)^a^	46.2 (25.4)^a^	0.605
TNF-alpha, ng/dl	13.1 (11.7)^a^	11.6 (12.1)^a^	15.3 (12.3)^a^	0.182
Erythrocytes, T/l	3.9 (2.3–5.6)	3.9 (2.5–5.6)	3.95 (2.3–5.5)	0.690
Hemoglobin, g/dl	11.55 (6.6–17.3)	11.55 (7.6–17.3)	11.75 (6.6–16)	0.953
Hematocrit, %	34.65 (19.9–50.2)	34.5 (25.0–50.2)	35.15 (19.9–49.2)	0.745
Platelet count, G/l	185 (23–637)	184 (29–436)	185 (23–637)	0.817
White blood count, G/l	*10.67 (3.2–38.5)*	*10.4 (3.2–26.39)*	*12.45 (3.58–38.5)*	*0.003*
Serum creatinine, mg/dl	1.28 (0.43–45.61)	1.19 (0.43–9.83)	1.72 (0.71–5.21)	0.957
BUN, mg/dl	24.95 (5.7–101)	23.55 (5.7–99)	39.5 (13–101)	0.002
Bilirubin, mg/dl	0.9 (0.33–16)	0.9 (0.33–16)	1.05 (0.35–5.7)	0.516
CRP, mg/dl	*2.25 (0.04–46.15)*	*1.86 (0.04–41.23)*	*5.57 (0.8–46.15)*	*0.016*
Fibrinogen, mg/dl	389 (6.44–1167)	387.5 (6.44–1099)	443.5 (114–1167)	0.363

*P*‑value refers to significance level of association with 30-days mortality using Student’s t‑test or Mann Whitney U test, as appropriate.

*p* ≤ 0.05 was considered statistically significant; data are presented as median

*BUN* blood urea nitrogen, *CRP* C-reactive protein, *MMP* matrix metalloproteinase, *TIMP* tissue inhibitor of metalloproteinase, *SAPS* Simplified Acute Physiology Score, *TNF* tumor necrosis factor

^a^interquartile range

^b^mean ± SD

^c^*p*-value alive vs. deceased

### MMPs and TIMPs levels on ICU admission and survival

Of all patients 24 (20.0%) died within 30 days. The patients’ gender had no statistically significant influence on 30-day mortality. On ICU admission 30-day survivors had significantly lower MMP‑9 and TIMP‑1 plasma levels than 30-day non-survivors (mean ± SD, pg/mL; 240.6 ± 206.8 vs. 419.6 ± 466.5 ng/mL, *p* = 0.009; 384.0 ± 298.2 vs. 719.9 ± 651.9, *p* < 0.001, respectively), whereas no significant differences were found for MMP‑2, TIMP‑2 and TNF-alpha levels (*p* = 0.126, *p* = 0.605, *p* = 0.182; respectively) at admission to ICU (Table 3). Furthermore, no correlation between ratios of MMPs/TIMPs (MMP-9/TIMP‑1, MMP-2/TIMP‑1, MMP-9/TIMP‑2 or MMP-2/TIMP‑2, *p* = 0.964, *p* = 0.060, *p* = 0.061 or *p* = 0.071, respectively) and survival was found.

**Table 3:** Unadjusted univariate logistic regression analysis assessing associations of MMP‑9, TIMP‑1 and SAPSII with 30-day mortality in all patients and in the cardiac disease subgroup

All patients		OR	95% CI	SD	*p*-value
MMP‑9. ng/ml		1.668	1.101–2.525	282.91	0.016
TIMP‑1. ng/ml		2.153	1.274–3.637	410.55	0.004
SAPS II score		3.830	2.127–6.897	21.95	<0.001
*Cardiac disease*					
MMP‑9. ng/ml		1.572	0.970–2.548	368.11	0.066
TIMP‑1. ng/ml		1.672	0.798–3.502	328.65	0.173
SAPS II score		3.527	1.554–8.008	21.47	0.003

Odds ratios (OR) are presented per standard deviation (1-SD) increase; *95%-CI* 95% confidence interval

*MMP* matrix metalloproteinase, *TIMP* tissue inhibitor of metalloproteinase, *SAPS* Simplified Acute Physiology Score

### MMPs and TIMPs on ICU admission and correlation with laboratory parameters

**Fig. 1:**
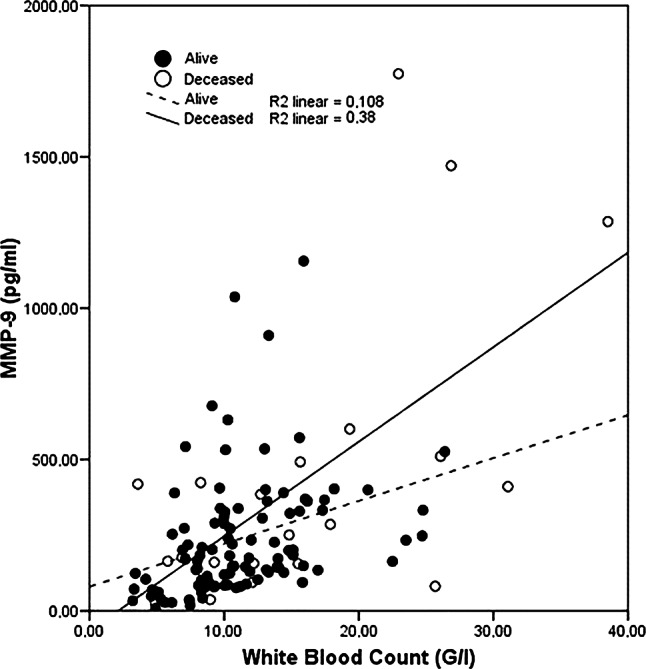
Correlation of MMP‑9 plasma levels with white blood cell count (*p* < 0.001)

The MMP‑9 levels showed an average correlation with white blood cell count (ρ = 0.492, *p* < 0.001) (Fig. 1).The measured plasma levels of MMP‑2, MMP‑9, TIMP‑1 and TIMP‑2 showed a positive correlation with proBNP levels (ρ = 0.309, *p* = 0.001; ρ = 0.184, *p* = 0.047; ρ = 0.511, *p* < 0.001; ρ = 0.203, *p* = 0.028; respectively). White blood cell count, blood urea nitrogen and C‑reactive protein were significantly higher in the non-survivor group compared to 30-day survivors (11.3 ± 4.8 G/l, 15.4 ± 9.0 G/l, *p* = 0.003; 30.4 ± 20.5 mg/dl, 45.8 ± 25.5 mg/dl, *p* = 0.002; 5.1 ± 7.4 mg/dl, 9.9 ± 12.6 mg/dl, *p* = 0.016; respectively) whereas the other investigated routine laboratory tests showed no differences.

### MMPs/TIMPs correlation with SAPS II score

**Fig. 2:**
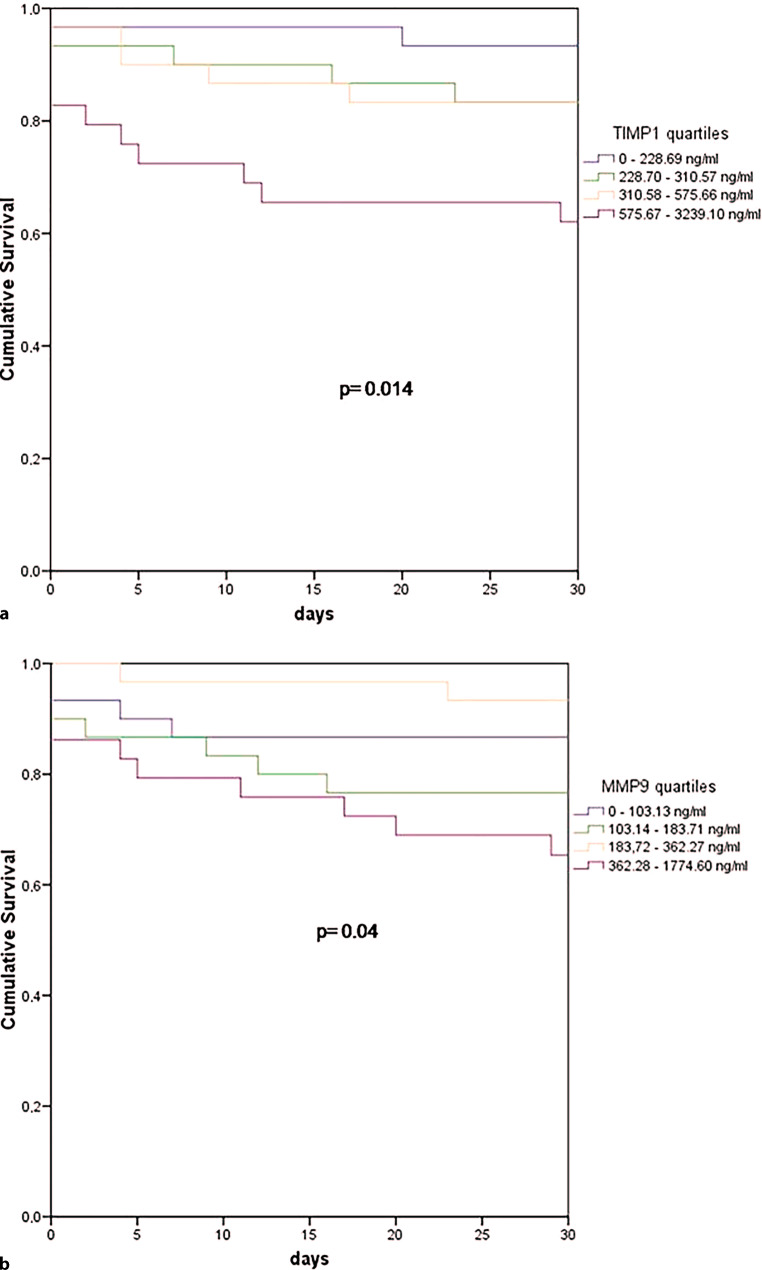
Kaplan-Meier survival estimates for TIMP‑1 (**a**)and MMP‑9 (**b**). Any cause 30-day ICU mortality according to TIMP‑1 and MMP‑9 plasma level quartiles

An average positive correlation between MMP‑9 and TIMP‑1 levels and the SAPS II score (ρ = 0.248, *p* = 0.007; ρ = 0.376, *p* < 0.001; respectively) was found, whereas MMP‑2 and TIMP‑2 levels showed no correlation with the SAPS II score using the Spearman correlation analysis. The ROC curve analysis showed area under the curve (AUC) for TIMP‑1 (AUC = 0.709, 95% CI = 0.584–0.834; *p* = 0.002) and SAPS II (AUC = 0.827, 95% CI = 0.731–0.924; *p* < 0.001) to predict 30-day survival; the same tendency was noted for MMP‑9 but did not reach statistical significance (AUC = 0.615, 95% CI = 0.479–0.752; *p* = 0.086). Fig. 2 shows Kaplan-Meier survival estimates for quartiles of MMP‑9 and TIMP‑1. Survival was significantly lower in the higher quartiles of MMP‑9 and TIMP‑1 when compared to lower MMP‑9 and TIMP‑1 values on admission (*p* = 0.04, *p* = 0.014 log-rank test over all four strata, respectively).

### MMPs/TIMPs and 30-day outcomes

In a logistic regression model, MMP‑9 was independently associated with 30-day survival in the subpopulation of patients with cardiac disease (*p* = 0.042). In a subgroup analysis, plasma MMP‑9 (*p* = 0.002) and TIMP‑1 (*p* = 0.01) levels upon admission to the ICU as well as SAPSII (*p* < 0.0001) were independent predictors for 30-day survival in the patient group with cardiac disease only. Results of univariate logistic regression analysis regarding the respective associations between MMP‑9, TIMP‑1, SAPSII and 30-day mortality (unadjusted OR per standard deviation increase, 95% CI and *p*-values) are presented for the entire study population and for the subgroup with cardiac disease in Table 3.

## Discussion

The results of this study indicate a prognostic significance of elevated TIMP‑1 levels and, to some extent, of MMP‑9 levels in a heterogeneous group of critically ill patients as emphasized by the significantly higher circulating plasma MMP‑9 and TIMP‑1 levels on ICU admission in patients who died within 30 days. The MMPs are known to play a role in the cytokine storm following systemic immune activation. They support immune cell migration, have vasoactive effects and can induce vascular leakage in severe illness [40, 41]. Circulating MMP‑9 concentrations are increased in the first few hours of systemic inflammation [42] and were shown to correlate with severity of associated organ injury in animals [43]. In our study, mean MMP‑9 concentrations correlated with established inflammatory markers, whereas MMP‑2 did not. In acute proinflammatory processes, MMP‑9 is released from granules of leukocytes [22] stimulated by cytokines such as TNF-alpha. Moreover MMP‑9 promotes leukocyte recruitment and cleaves TNF‑alpha into its active form [11]. In the early phase after acute myocardial infarction MMP‑9 is secreted by neutrophils and potentially by macrophages [66]; it can modulate white blood cell function and further create a positive feedback loop for neutrophil activation and chemotaxis via IL‑1 beta (interleukin), i.e. lymphocyte activating factor and IL‑8 [67]. Consistently, white blood cell count and TNF‑alpha concentration significantly correlated with MMP‑9 levels in our patients.

Previous studies investigating the relation between MMPs and mortality in larger cohorts of critically ill patients were mainly conducted in patients suffering from sepsis [16, 18, 42, 47]. Our study sample comprised patients with heterogeneous underlying critical conditions, of which only one condition was sepsis. In line with our findings Nakamura et al. [47] reported significantly higher early MMP‑9 levels in patients who died of (septic) shock when compared to survivors. Hoffmann et al. also noted elevated MMP‑9 in patients who died of sepsis; however, their results did not reach a level of statistical significance [16]. Elevated MMP‑9 levels have further been reported in several other severe conditions such as severe brain injury [14] and stroke [45]. Plasma MMP‑9 activity has further been described as potentially predictive for lung injury and the development of ARDS (acute respiratory distress syndrome) in critically ill patients [46]. In our study sample, when performing subgroup analysis, MMP‑9 levels were an independent predictor of survival in patients with underlying cardiac disease. The MMP‑9 levels and associated inflammatory cytokines were previously related to acute cardiovascular events [44], disease severity in chronic heart failure and adverse outcomes in these patients [37]. Our findings are also in line with a recent report by Lahdentausta et al. supporting MMP‑9 as an early stage biomarker of poor outcome in cardiovascular disease potentially reflecting atherosclerotic plaque rupture and myocardial tissue destruction [49]. The MMP‑9 levels further correlate with left ventricular ejection fraction and were associated with perioperative myocardial injury in coronary bypass grafting [48], which was an underlying diagnosis in some of our patients. To our knowledge, this is the largest published study to date, demonstrating the prognostic value of MMP‑9 levels in a heterogeneous group of critically ill patients admitted to intensive care.

Further a clear association between elevated TIMP‑1 levels and 30-day mortality could be shown in our study population. The TIMP‑1 levels are known to be elevated in inflammatory and ischemic events; they are induced by proinflammatory and profibrotic stimuli and are to some extent independent of MMPs regulation [50]. Our findings are consistent with previously published study results investigating the impact of TIMP‑1 levels on mortality in critical care settings. Lorente et al. reported elevated circulating TIMP‑1 levels on admission in sepsis non-survivors and showed higher TIMP-1/MMP‑9 ratios at days 1, 4 and 8 to predict mortality in a large multicenter study [17, 18]. They concluded that a higher TIMP-1/MMP‑9 ratio was associated with severity, coagulation state, circulating cytokine levels and mortality and proposed it as an outcome biomarker of sepsis. Similarly, although in a smaller sample size, Hoffmann et al. described TIMP‑1 as an efficient prognostic marker for fatal outcome in septic patients [16]. Analogous findings were reported for sepsis-associated organ dysfunction after major abdominal surgery by another study group [51]. Taking into account positive and negative predictive values, however, Serrano-Gomez et al. found neither MMP‑9, MMP‑2, TIMP‑1, TIMP‑2 nor their respective ratios to have significant predictive values in mortality of sepsis patients [52]. The TIMP‑1 has been associated with survival in several other critical diseases, including traumatic brain injury [53, 54], cerebral infarction [55], graft versus host disease [56] and acute respiratory failure [57]. Possibly upregulated as a reaction to MMP release in the cytokine storm, elevated TIMP‑1 levels could favour microcapillary thrombosis and fatal multiple organ dysfunction, as previously proposed [17]. Interestingly, in a large longitudinal study, TIMP‑1 was a strong predictor of all-cause 10-year mortality, with most studied patients dying of cardiovascular disease [58]. Furthermore, higher TIMP‑1 levels were reportedly associated with cardiovascular events in a Chinese follow-up study of patients with coronary heart disease [59]. Episodes of ventricular tachyarrhythmia, potentially involved in sudden cardiac death, were associated with higher MMP‑9 levels, and particularly MMP-9/TIMP‑1 ratios in heart failure patients [60]. Disease progression in coronary artery disease was previously associated with an increasing MMP-9/TIMP‑1 ratio in circulating CD14+ monocytes. Disparity between TIMP‑1 and MMP‑9 levels was also shown to contribute to adverse events and mortality in heart failure patients [37]. In our patients, MMP-9/TIMP‑1 ratios were not statistically associated with survival. Other studies reporting prognostic relevance of MMP-9/TIMP‑1 ratios in a comparable setting of myocardial infarction [61] were conducted in serum, which might explain the disparity our findings [62]. Also, TIMP‑1 is involved in MMP-2/MMP-9-independent mechanisms, which might further explain the independent predictive value of TIMP‑1 in major adverse cardiovascular events, as proposed by Lindsey et al. [63]. In this context, levels of TIMP‑1 (and TIMP-2), correlated with acute phase proteins (CRP [C reactive protein], fibrinogen) in our patients, possibly indicating an aligned regulation. Taken together, our study provides further support for the prognostic potential of TIMP‑1 and contributes to expanding the predictive value of early TIMP‑1 levels to 30-day survival in a heterogeneous group of critically ill patients. Here survival significantly correlated with TIMP‑1 and MMP‑9 levels in line with known markers of systemic inflammation (WBC, CRP) and organ decompensation (proBNP, BUN). The excellent correlation between the well-established clinical score of survival, SAPSII, and TIMP‑1, but also MMP‑9 levels, further accentuates the significance of our main findings.

Concerning MMP‑2 and TIMP‑2 levels, however, no correlation with respect to the endpoints could be demonstrated. In agreement with our results, other authors consistently found elevated MMP‑9 levels in patients with acute systemic inflammation [22, 24, 35], whereas MMP‑2 levels did not differ between patients and controls in any of these studies. The use of MMP‑2 has been described as a possible long-term prognostic marker in heart failure patients by George et al. [34]. The authors reported an association between elevated MMP‑2 levels and mortality over a 24-month period. Higher plasma MMP‑2 levels in type 1 diabetes patients were further associated with cardiovascular events and all-cause mortality in a 12-year follow-up study [64]. In cases of severe Chagas cardiomyopathy MMP‑2 levels added to predictive value of other biomarkers with respect to 1‑year mortality [65]. Even though the role of MMP‑2 remains undisputed in cardiovascular disease [5, 22, 23] it appears to be more relevant as a predictor of long-term outcomes whereas MMP‑9 has been more frequently reported in (sub)acute systemic responses following cardiac and non-cardiac events [34, 35], which is in line with our findings.

In summary, 30-day survival was predicted by higher MMP‑9 and TIMP‑1 plasma levels on ICU admission in critically ill patients and TIMP‑1 and also MMP‑9 were significantly correlated with SAPS II, a scoring system specifically designed to estimate disease severity and predict fatal outcomes in ICU patients, further supporting our findings.

## Limitations

There are some limitations regarding the generalizability of our results in ICU patients and the outcome. Firstly, despite the large study sample, the size of our subgroups with non-cardiac diseases, the smallest of which was our sepsis subgroup, could be considered insufficient for general comparison between outcomes in cardiac and non-cardiac disease patients. It cannot be excluded that larger studies concerning MMP and mortality, conducted in respiratory failure patients, might find trends which were not detected in our setting. Sepsis, however, as well as infection-associated mortality and organ dysfunction have been extensively studied in this context with outcomes comparable to the findings in our subgroup. Secondly, our patients’ diagnoses were determined on admission to ICU and do not necessarily indicate the final causes of death. Thus, our findings cannot be definitely related to specific organ system failures without autopsy and histopathological results, which were not included for this study. Lastly, as our aim was the prognostic relevance of MMP and TIMP levels for short-term mortality, they were only assessed at one singular but critical time point, i.e. an acute setting leading to ICU admission. Follow-up trends in MMP and TIMP levels and long-term mortality were not addressed in this study.

## Conclusion

The prognostic significance of (early) TIMP‑1 levels has been extended to a heterogeneous group of critically ill patients and further support the predictive value of MMP‑9 levels on short-term mortality, especially in patients with underlying cardiac disease. These findings indicate a value of MMP‑9 and TIMP‑1 as prognostic markers and also as potential candidates for a multibiomarker approach and therapeutic targets in systemic inflammation and in settings of acute organ failure.
